# Exploratory In Vitro Evaluation of Maternal–Infant *Bifidobacterium* Strains for Microbiota Modulation in a Pediatric Cystic Fibrosis Context

**DOI:** 10.3390/microorganisms13112523

**Published:** 2025-11-02

**Authors:** Maria Esteban-Torres, Isabel Blanco, Andrea Asensio-Grau, Nuria Ruiz, Manuel Bernabeu, Joaquim Calvo-Lerma

**Affiliations:** 1 Institute of Agrochemistry and Food Technology, Spanish National Research Council (IATA-CSIC), 46980 Paterna, Valencia, Spain; 2 Department of Preventive Medicine and Public Health, Food Science, Toxicology and Forensic Medicine, Faculty of Pharmacy and Food Science, Universitat de València, Avda. Vicent Andrés Estellés, 46100 Burjassot, Valencia, Spain

**Keywords:** probiotics, *Bifidobacterium*, gut microbiota, SCFAs, in vitro fermentation, cystic fibrosis

## Abstract

This study explores the potential of novel *Bifidobacterium* isolates as targeted probiotic supplements for children with cystic fibrosis (CF), a condition often associated with gut dysbiosis. Five strains of *Bifidobacterium* genus (*B*. *animalis* IATA01, *B*. *pseudocatenulatum* IATA35, *B*. *longum* IATA02, *B*. *bifidum* IATA13, and *B*. *longum* IATA05) isolated from maternal–infant fecal samples were assessed in vitro following the FAO/WHO guidelines. Their probiotic potential was evaluated through simulated gastrointestinal digestion in the CF context, their adhesion to mucin, and their carbohydrate fermentation capacity. Additionally, their impact on colonic microbiota modulation was analyzed using static in vitro colonic fermentation with fecal inocula from four pediatric patients with CF to assess the presence of different bacterial groups associated with dysbiosis via qPCR and short-chain fatty acid production by GC-MS. Three strains (IATA01, IATA35, and IATA05) demonstrated survival after gastrointestinal digestion, with IATA01 exhibiting the highest adhesion to mucin but limited carbohydrate fermentation capacity. All strains increased the *Bifidobacterium* levels after colonic fermentation, while their effects on reducing pathogenic groups and promoting beneficial bacteria such as *Akkermansia* and *Faecalibacterium* varied depending on the strain and the individual inoculum. These findings highlight the strain-specific effects of *Bifidobacterium* and evidence a specific impact on colonic microbiota, depending on the composition of the basal inoculum, highlighting individual-specific responses.

## 1. Introduction

Dysbiosis, defined as a reduction in microbial diversity along with an imbalance between beneficial and pathogenic bacteria, is a challenge associated with many diseases such as cystic fibrosis (CF) [1,2]. The consequences of dysbiosis encompass the generation of pro-inflammatory microbial metabolites in the colonic environment, which are behind the origin of several diseases or complications [1,3]. Several strategies have been proposed to counteract dysbiosis, including specific dietary patterns and nutritional counselling, as well as the use of the biotics family, including prebiotic and probiotic supplements. Among these, probiotics—live microorganisms that, when administered in adequate amounts, confer a health benefit on the host [4]—have attracted interest for their potential to restore gut microbial balance.

Concerning probiotics, bifidobacteria have been largely used as probiotics, given the fact that they have proven health benefits [5]. Bifidobacteria are gut commensal bacteria that dominate the intestinal microbiota during early infancy, especially in breast-fed infants; however, they remain consistently present across all age groups [6]. In this sense, some *Bifidobacterium* strains have a protective effect against diarrhea, as well as reducing side effects of antibiotic treatment [7]. Others showed improved inflammatory status in obese children with insulin resistance [8], among many other reported health benefits. However, not all *Bifidobacterium* strains exert the same beneficial effects; therefore, the probiotic potential of specific strains should be tested prior to their application as probiotics [5].

CF is characterized as dysbiosis in the gut microbiota as a consequence of combined factors, including lipid maldigestion (unabsorbed fats reaching the colon), an acidic intestinal environment due to reduced pancreatic bicarbonate secretion, and the continuous use of antibiotics to treat lung disease. Thus, children with CF exhibit specific alterations in their gut microbiota in terms of diversity, composition, and functionality. Lower alpha-diversity is reported in infants, as well as increased populations of potential pathogenic bacteria, such as members of *Enterobacteriaceae*, *Streptococcus*, and *Enterococcus*, among others [2]. In contrast, the relative abundance of various commensal and potentially beneficial bacteria, including *Bifidobacterium*, *Akkermansia*, and butyrate-producers such as *Faecalibacterium* members, was typically decreased [2,9]. The multifactorial complexity of the disease (pancreatic insufficiency, use of antibiotics, increased respiratory rate and energy expenditure) contributes to the deterioration of nutritional status in affected children [10]. Current strategies to improve it include probiotic supplementation; however, the limited number of studies that are currently available does not yet support the recommendation of specific strains or patterns for supplementation. Emerging evidence suggests that personalized approaches, tailored to the individual microbiota profiles of patients with CF may be more effective in guiding probiotic interventions [11]. Therefore, exploring the potential of *Bifidobacterium* strains as probiotics in the context of CF microbial modulation could be a valid approach to try to improve dysbiosis, but up to date, there is no evidence in CF.

This study aimed to identify novel *Bifidobacterium* strains with probiotic potential by assessing their functional capabilities relevant to modulating colonic microbiota in paediatric patients with CF.

## 2. Materials and Methods

### 2.1. Bifidobacterium Strains and Growth Conditions

The five *Bifidobacterium* strains used in this study belong to the bacterial collection from the group of Maria Carmen Collado at IATA-CSIC, Spain. The *Bifidobacterium* strains were isolated from five healthy volunteer mothers and their babies from the MAMI (MAternal MIcrobes) cohort. The MAMI cohort is a prospective mother–infant cohort in the Spanish Mediterranean area, as described elsewhere [12]. Given our exploratory objective and the pediatric setting, we selected five maternal–infant *Bifidobacterium* isolates representing phylogenetic and ecological diversity within the genus and known early-life colonisers. This design prioritised breadth of strain behaviours under CF-relevant constraints rather than pre-selection for specific glycan pathways.

All participants received oral and written information about this study and written consent was obtained. This study was approved by the hospital ethics committees (Hospital Clínico Universitario de Valencia) and registered on clinicaltrials.gov with registration number NCT03552939.

The bacteria were isolated as previously described [13]. For taxonomic identification, a fragment of approximately 898 nt of the 16S rRNA gene was amplified by PCR using the primers 27F (5′-GAGTTTGATCMGGCTCAG-3′) and 924R (5′-CTTGTGCGGGYYCCCGTCAA-3′) to identify the strains taxonomically. PCR mix was prepared with MgCl_2_ 50 mM, DMSO, dNTPs 10 mM, primers 27F 10 µM and 924R 10 µM, Taq polymerase 5 U/µL, reaction buffer for NZY Taq II Pol (Nztech, Auckland, New Zealand), and molecular-grade water. The reaction parameters used were initial denaturation at 94 °C for 4 min and 30 cycles at 92 °C for 30 s, 65 °C for 30 s, 72 °C for 30 s, and 72 °C for 5 min. Amplicons were sent to Eurofins Genomics Europe Shared Services GmbH for Sanger sequencing.

In order to identify the bacterial isolates at the species level, 16S sequence data were analysed by nucleotide BLAST at the National Center for Biotechnology Information (http://www.ncbi.nlm.nih.gov, accessed on 14 May 2023), using the reference RNA sequences database (RefSeq/rRNA). The highest identity (≥99% of identity and ≥98% query cover) was selected to assign a particular species (of note, 16 rRNA supports species-level assignment only, with thresholds used; not subspecies).

Isolated *Bifidobacterium* strains (conserved in 20% glycerol at −80 °C) were grown on agar o liquid MRS (BD Difco™, Thermo Fisher Scientific, Alcobendas, Spain) media supplemented with 0.05% cysteine. Firstly, bifidobacterial strains were grown on solid media (MRS agar plates supplemented with 0.05% cysteine) and incubated for 48–72 h at 37 °C in anaerobic conditions using anaerobic jars with AnaeroGen sachet (Thermo Fisher Scientific, Alcobendas, Spain). Then, isolated colonies were grown on liquid medium MRS supplemented with 0.05% cysteine an incubated in the same conditions for 16–24 h.

### 2.2. Functionality Screening

#### 2.2.1. Survival in In Vitro Simulated Gastrointestinal Conditions

The bacterial survival in the gastrointestinal conditions was assessed by subjecting the bifidobacterial strains to the in vitro digestion neonatal model described by Brodkorb et al. (2019) [14]. The bifidobacterial strains were grown in MRS supplemented with 0.05% cysteine for 16 h. Then, the culture was centrifugated and washed once in 1.8 mL PBS, and the bacterial pellet was resuspended in 1.8 mL PBS. Then, the OD_595_ was measured and 1 × 10^8^ cfu/mL of bacterial suspension was inoculated in the gastric or intestinal solutions (1 mL, 1/1 *v*/*v*). This experiment was performed in duplicate. The gastrointestinal digestion conditions were the following: the simulated gastric fluid (SGF) was added in 1:1 (*v*/*v*) ratio to the bacterial suspension, pH was set at 3 with 1M HCl, and the temperature was set at 37 °C. The enzyme concentrations of pepsin and lipase from rabbit gastric fluid in the SGF were 2000 U/mL and 60 U/mL, respectively. After 120 min, the simulated intestinal fluid (SIF) was added to gastric digesta in a 1:1 (*v*/*v*) proportion, pH was established at 6.6, and temperature remained at 37 °C for 120 min. The SIF contained pancreatin (trypsin activity = 100 U/mL) and 1 mM bovine bile extract. Lower intestinal pH and bile salts concentration with respect to Brodkorb et al.’s (2019) [14] protocol were simulated, so that the intestinal conditions were closer to those present in children with CF [15,16], as previously done in similar studies [17,18]. The oral phase was omitted in this model, as the study sample was not a solid food [14].

For the survival analysis, aliquots of 1 mL were obtained at each time point (gastric initial, gastric final, intestinal initial, intestinal final) and diluted 100 times in 0.85% NaCl solution. The aliquots (200 µL) were stained with 2 µL Live/Dead BacLight bacterial viability kit (Molecular Probes, Eugene, OR, USA) and incubated for 15 min at room temperature in darkness following the manufacturer’s instructions. Live cells (unstained) and live and dead cells (isopropanol-treated), stained as before, were used to adjust the flow cytometer parameters for an optimal flow rate and events count. Bacterial quantification was carried out in a MACSQuant 16 (Miltenyi Biotec, Bergisch Gladbach, Germany). The data were analysed with FCS express version 5 flow cytometry software.

#### 2.2.2. Carbohydrates Growth Capacity

The growth of *Bifidobacterium* strains on several carbohydrates, which were the sole carbohydrate source, using glucose as a positive control, were determined in medium minimum MRS (mMRS) prepared from first principles [19] and supplemented with 1% (*w*/*v*) of each carbohydrate and 0.05% cysteine in 96-well plates (200 µL final volume). The carbohydrates tested were D-(+)-glucose (Sigma-Aldrich, Taufkirchen, Germany), D-(+)-lactose (AppliChem, Darmstadt, Germany), Inulin from chicory (Sigma-Aldrich, Germany), fructooligosaccharides (FOS) from chicory (Sigma-Aldrich, Germany), D-galactose (Boehringer, Germany), D(-)-fructose (Sigma-Aldrich, Germany), and D-(-)-ribose (Sigma-Aldrich, Germany). The carbohydrates were prepared at 10% (*w*/*v*) in water and sterilized by 0.2 µm filters. The inclusion of some human milk oligosaccharides was carried out because of recent evidence that they are potential prebiotics for older children and adults [20,21].

Inulin and FOS were prepared in mMRS at a final concentration of 1% (*w*/*v*) and autoclaved at 121 °C for 15 min. The human milk oligosaccharides including lacto-N-tetraose (LNT), lacto-N-neotetraose (LNnT), 2′-fucosyllactose (2FL), 2′-fucosyllactose/difucosyllactose mixture (2FL/DFL), 3′-sialyllactose (3SL), and 6′-sialyllactose (6SL) were kindly provided by DSM, Denmark. The human milk oligosaccharides were added after autoclaving mMRS and filtered using a 0.22 µm filter. For bacterial inoculum, the overnight cultures on MRS were centrifugated at 3385× *g* for 10 min at 4 °C. The pellets were washed with 1.8 mL of PBS once and centrifugated as before. The pellets were resuspended in 1.8 mL PBS and the OD_595_ was adjusted to 0.5 in PBS (final OD_595_ was 0.05). The plates were incubated at 37 °C for 24 h in anaerobic conditions and the final OD_595_ was measured in a Spectrostar Nano spectrophotometer (BMG Labtech, Ortenberg, Germany).

#### 2.2.3. Adhesion to Mucin

The capability of adhesion to mucin of the bifidobacterial strains was evaluated following the previously described method [22] with some modifications. Plates were covered with 500 µg/mL mucin (porcine stomach, Sigma-Aldrich, Germany) in 50 mM carbonate/bicarbonate and left overnight at 4 °C. Wells without mucin were included as the control. Plates were washed with PBS and blocked for 1 h with PBS plus 1% Tween 20. Then, 100 µL of each strain was added to each well in PBS adjusted to an OD_595_ nm of 1 (1 × 10^8^ cfu/mL) and plates were incubated for 2 h at 37 °C. Non-adhered cells were removed by washing three times with PBS plus 0.05% Tween 20 and the plates were dried at 55 °C. Adhered cells were stained with crystal violet 1 mg/mL for 45 min. After six washes with PBS, the colorant was liberated with 10% acetic acid (100 µL/well) for 45 min and the absorbance at 595 nm was determined in a plate reader. Adherence to mucin was considered as the difference in absorbance at 595 nm between the wells with mucin and those without mucin.

### 2.3. Simulation of Colonic Fermentation of Infants with Cystic Fibrosis

A static in vitro colonic fermentation model was established to evaluate the potential of the probiotic strains in modifying the basal colonic microbiota and metabolic activity of 4 children with CF. The model consisted of incorporating a fecal inoculum into a glass vial containing a basal culture medium [23], adding the bacterial strain, and incubating in anaerobiosis conditions at 37 °C for 24 h. Four different colonic environments were simulated by using the fecal inocula from 4 children with CF, which presented different individual characteristics that could have a different and person-specific impact on their microbiota, such as the use of antibiotics [24], pulmonary infections, CFTR modulator therapy [2], or age [25] (Table 1).

The fecal inocula were obtained by mixing 3 g of a fresh fecal sample with PBS 0.9% in a proportion of 1:10 (*w*/*v*) and centrifuging (5 min, 1504× *g*, 4 °C). Then, 0.1 mL of the supernatant was incorporated into a glass vial containing 1.2 mL of a basal culture medium that was previously described [26]. The resulting mixture was kept overnight in anaerobiosis conditions and at 37 °C to allow for microbiota stabilization in the new in vitro environment. After this time, 0.5 mL of PBS containing the bacterial strain (1 × 10^8^ cells/mL) was added to start the simulation of colonic fermentation over a 24 h period (anaerobiosis, 37 °C). The process was reproduced by placing the glass vials into an anaerobiosis container incorporating anaerobiosis devices (AnaeroGen, Thermo Scientific, Alcobendas, Spain) and then into a thermostatic chamber at 37 °C.

After 24 h of simulated colonic fermentation, final aliquots of 0.75 mL were centrifuged (1504× *g*, 10 min, 4 °C). The pellet was used to extract DNA and quantify bacterial groups, and the supernatant was kept for SCFA determination.

The changes in the target bacterial groups were expressed as the difference between the number of copies obtained after simulated colonic fermentation and the number of copies identified in the faecal inoculum, both expressed as the logarithm.

The fecal inocula were obtained from 4 pediatric patients with CF in follow-up at the Pediatric Cystic Fibrosis Unit of La Fe University and Polytechnic Hospital, after receiving written informed consent by the parents and the children aged >12 years old. This study was approved by the ethics committee with registration number 2021-111-1, and conducted according to the Declaration of Helsinki.

### 2.4. Analysis of Colonic Microbiota

To characterize changes in colonic microbiota, different bacterial groups were determined by quantitative PCR (qPCR), following a previously described procedure [26], including total bacteria, *Bifidobacterium* genus, *Bacteroides* genus, *Faecalibacterium prausnitzii*, *Akkermansia muciniphila*, enterobacteria group, and *Streptococcus* genus (primers indicated in Supplementary Table S1). These bacterial groups are considered key indicators of standard or altered colonic microbiota [8].

First, DNA was extracted from the pellet fraction obtained after simulated colonic fermentation (approx. 100–200 mg) using the Master-Pure DNA extraction Kit (Epicentre, Madison, WI, USA), applying the manufacturer’s instructions with the following modifications: samples were treated with lysozyme (20 mg/mL), lyticase (2.5 U/mL), and mutanolysin (5 U/mL) for 60 min at 37 °C and then disrupted with 3 µm diameter glass beads, which was followed by 2 cycles of 30 sec at 6 m/s in a FastPrep 24-5G Homogenizer (MP Biomedicals) bead beater [27]. DNA concentration was measured using Qubit^®^ 2.0 Fluorometer (Life Technology, Carlsbad, CA, USA).

Then, qPCR was conducted to quantify the bacterial groups previously indicated. The qPCR amplification and detection were performed in duplicate on a LightCycler 480 Real-Time PCR System (Roche Technologies, Mannheim, Germany). Each reaction mixture of 10 µL was composed of SYBR Green PCR Master Mix (Roche), 0.5 µL of each primer (concentration 10 mmol/L), and 1 µL of DNA template (Supplementary Table S1). The bacterial concentration in each sample was calculated by comparing the Ct values obtained from standard curves. To create a standard ladder for 16S rRNA gene copy number extrapolation for each bacterial group, genomic DNA were amplified, purified, and quantified as previously described [26]. Standard curves were obtained by serial 10-fold dilutions of purified standard DNA fragments, corresponding to 16S rRNA gene regions described above, covering the range 10–10^9^ gene copies/mL, which were run in parallel and used to extrapolate the number of 16S rRNA gene copies in the microbiota samples. Each dilution was analysed in triplicate, and Ct values were plotted against the log of the known copy number. The curves showed high linearity (R2 > 0.99), ensuring a robust quantification of the bacterial groups in the microbiota samples.

Data were expressed as total number of copies per mL of sample at baseline and after 24 h of colonic fermentation, and also as the logarithm. Then, changes after 24 h of colonic fermentation with respect to baseline values in each inoculum were expressed as the difference in the logarithm of the number of copies.

### 2.5. Determination of Short-Chain Fatty Acids (SCFA)

After centrifugation of colonic digesta tubes, the supernatant was used to determine the production of SCFA by gas chromatography-mass spectrometry (GC-MS), following the protocol by Eberhart and collaborators with some modifications [28]. Briefly, 200 μL aliquots were mixed with 800 μL of the internal standard extraction solution (3-methylvaleric acid) and vortexed. Then, 1 mL of diethyl ether was incorporated along with a spoon tip of Na_2_SO_4_ to remove any traces of water in the extract before injection, allowing for 10 min of reaction, and then centrifuged 2000× *g* for 5 min. The supernatant was, at this point, injected in the Agilent GC7890B-5977B GC-MS with a multipurpose sampler (Gerstel MPS) with an Agilent DB-FATWAX column (30 m × 0.25 mm × 0.25 μm) operated in split mode (20:1). The oven temperature programme was 100 °C for 3 min, with the temperature being ramped to 100 °C at a rate of 5 °C min^−1^, to 150 °C for 1 min, to 200 °C at a rate of 20 °C min^−1^, and then finally being held at 200 °C for 5 min. Helium was used as carrier gas at a flow rate of 1 mL min^−1^; inlet temperature of 250 °C; injection volume: 2 μL. For the quantification of the SCFAs, 3-methylvaleric acid was used as an internal standard at a concentration of 5 mM and standard curves for acetate, propionate, isobutyrate, and butyrate were prepared. Agilent MassHunter Workstation Software version B.09.00 was used for quantitative analysis.

### 2.6. Statistical Analysis

All the data were summarized by mean and standard deviation. Two-way ANOVA analyses were applied to assess differences between bacterial strains in the different study outcome variables: survival of gastrointestinal digestion (compared to baseline values), carbohydrate fermentation capacity (compared to MRS as control), adhesion to mucin (compared to PBS as control), changes in colonic microbiota (compared to baseline microbiota), and production of SCFA (compared to baseline values). The GraphPad Prism software (version 9.5.1) was used to conduct the statistical analyses.

## 3. Results

### 3.1. Identification of Selected Bifidobacterium Isolates

Several *Bifidobacterium* strains were isolated from mother–infant feces and identified at the species level. Among these, five representatives were selected for further characterization and designated as *B*. *animalis* IATA01, *B*. *pseudocatenulatum* IATA35, *B*. *longum* IATA02, *B*. *bifidum* IATA13, and *B*. *longum* IATA05 (Table 2). These strains were chosen to represent phylogenetic and potential functional diversity withing the genus. Most of the strains were obtained from maternal faeces, given the fact that the mother is the main source of *Bifidobacterium* for infant gut microbiota [29]. While identification was performed at the species level, two strains of *B*. *longum* species showed a sequence identity with different subspecies and both were included, although this methodology does not allow sub-species resolution. One *B*. *longum* strain from infant faeces (*B*. *longum* IATA02) was selected for potential different performance with a *B*. *longum* strain from maternal origin (*B*. *longum* IATA05).

### 3.2. Functional Capacities of the Strains

#### 3.2.1. Survival to Gastrointestinal Digestion

**Figure 1:**
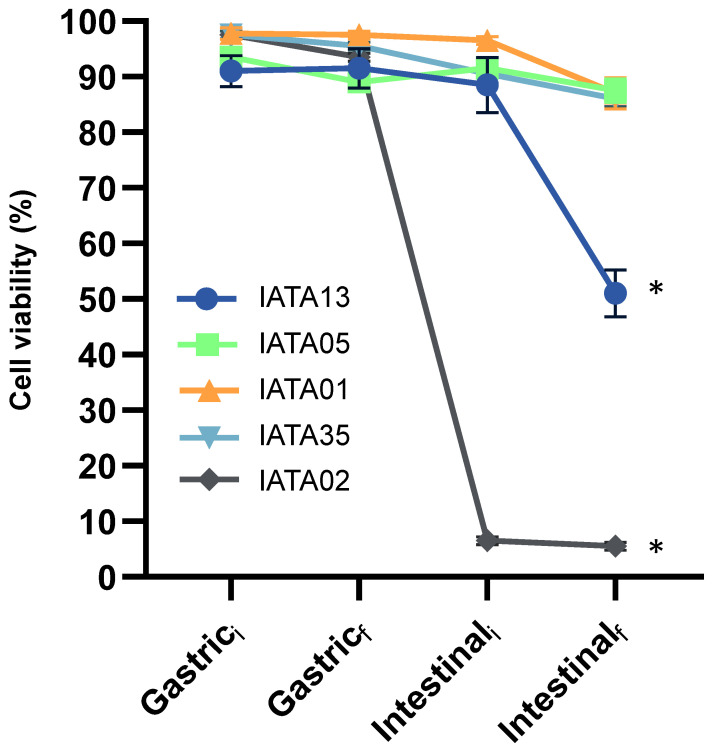
Percentage of live bacteria along the simulated gastric and intestinal stages of digestion, at time 0 min (i) and time 120 min (f), as measured by MACSQuant 16 and analysed by FCS express v5. Values represent the mean percentage and standard deviations for live populations of two independent replicates. * Statistically significant difference compared to Gastric_i_. Strain codes: IATA01, *Bifidobacterium animalis*; IATA35, *Bifidobacterium pseudocatenulatum*; IATA02, *Bifidobacterium longum*; IATA13, *Bifidobacterium bifidium*; IATA05, *Bifidobacterium longum*.

The survival of gastrointestinal conditions is an important factor for probiotic characterization. The exposure to gastric and small intestine digestion conditions led to variable effects on bacterial strains survival. According to Figure 1, *B*. *longum* IATA05, *B*. *animalis* IATA01, and *B*. *pseudocatenulatum* IATA35 were able to survive both stages of digestion, at least close to 90%, with this extent being significantly higher than that of the other two strains. In particular, *B*. *animalis* IATA01 remained unaltered through the process, but the 2 h duration of small intestine digestion led to a 10% decrease in its survival, while, in the other two strains, the decrease was more evenly distributed. The other two strains, *B*. *bifidum* IATA13 and *B*. *longum* IATA02, showed different tendencies, with their final extents of survival being below 50%. Focusing on *B*. *longum* IATA02, a 5% decrease in viability was observed after 120 min of the gastric stage, and the incorporation of the intestinal fluids (intestinal stage t = 0 min) caused a sharp decrease in survival, which was below 10%. In the case of *B*. *bifidum* IATA13, the incorporation of the gastric fluid (gastric stage t = 0 min) was enough to reduce survival to 90%, but this value was maintained until the beginning of the intestinal stage. However, at the end of that stage, survival was reduced to 50%. The strain that showed the worst survival of gastrointestinal digestion was *B*. *longum* IATA02, since less than 10% survived in the intestinal conditions.

#### 3.2.2. Growth Capacity on Carbohydrates

**Figure 2:**
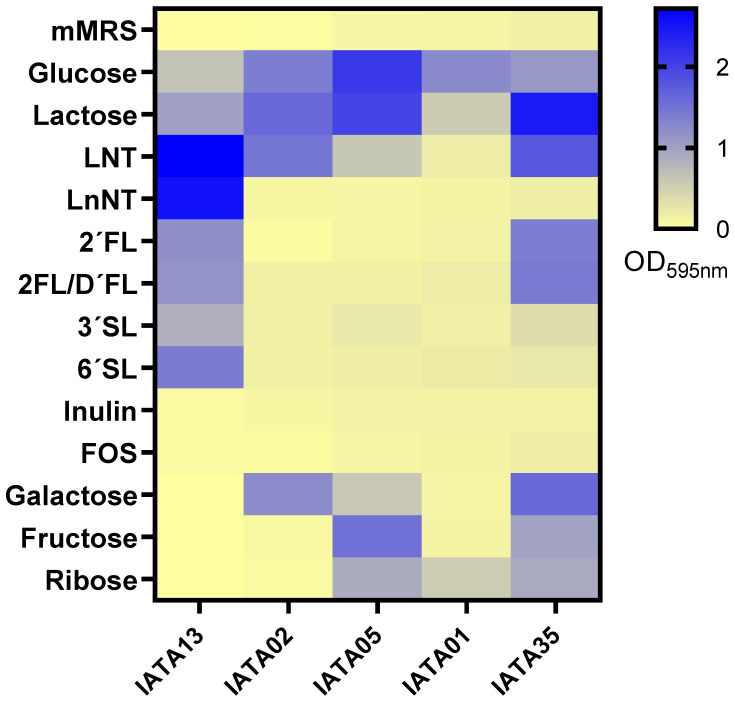
Carbohydrate utilization of the tested strains of *Bifidobacterium* on mMRS medium supplemented with 1% (*w*/*v*) of different carbohydrates: lacto-N-tetraose (LNT), lacto-N-neotetraose (LnNT), 2′fucosyllactose (2′FL), mix 2′fucosyllactose and difucosyllactose (2′FL/D′FL), 3′-sialyllactose (3′SL), and 6′-sialyllactose (6′SL). The culture of the strain on mMRS was used as a negative control. The figure represents the logarithm of final OD_595_ after 24 h of incubation at 37 °C in anaerobic conditions. Strain codes: IATA01, *Bifidobacterium animalis*; IATA35, *Bifidobacterium pseudocatenulatum*; IATA02, *Bifidobacterium longum*; IATA13, *Bifidobacterium bifidium*; IATA05, *Bifidobacterium longum*.

The colonization capabilities of probiotic strains are influenced by the utilization of certain carbohydrates from the diet or the host. The bifidobacterial strains were tested for their capability to grow on 13 different carbohydrates, including six human milk oligosaccharides (HMOs) (Figure 2). The strains showed differences in carbohydrate utilization. The strain *B*. *pseudocatenulatum* IATA35 grew on most of the tested carbohydrates, efficiently utilizing glucose, lactose, LNT, 2′FL, galactose, fructose, and ribose. In contrast, the strain *B*. *animalis* IATA01 only grew efficiently on glucose, lactose, and ribose. The strain *B*. *bifidum* IATA13 efficiently utilized all the HMOs tested (LNT, LnNT, 2′FL, 3′SL, and 6′SL) for growing, while the *B*. *longum* strains *B*. *longum* IATA02 and *B*. *longum* IATA05 and *B*. *animalis* IATA 01 did not grow on most of the carbohydrates, and none of the strains could grow on inulin or FOS.

#### 3.2.3. Mucin Adhesion Capacity

**Figure 3:**
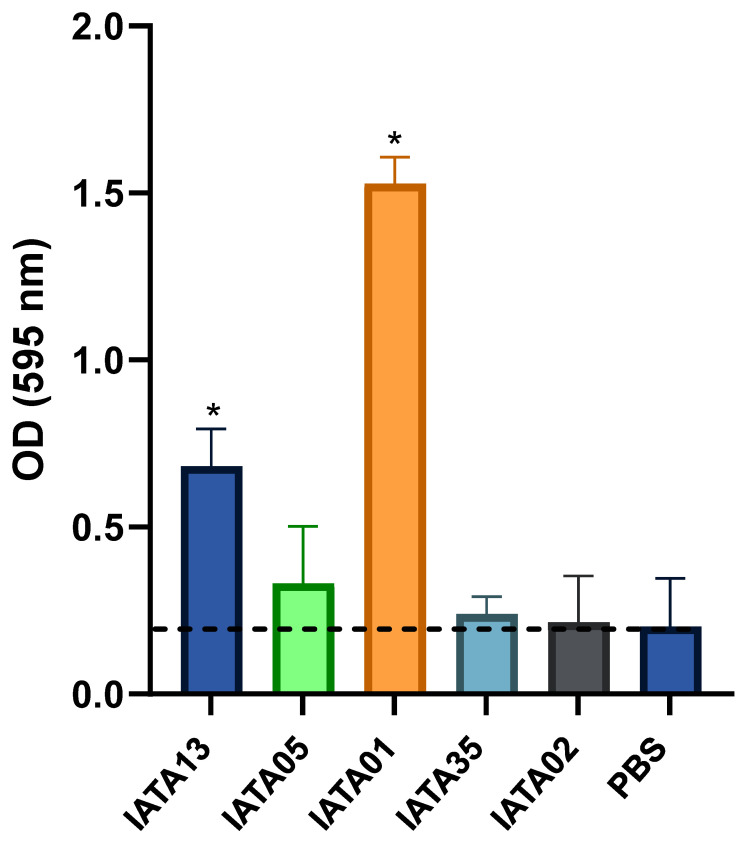
Adhesion of *Bifidobacterium* strains to mucin. The strains were incubated with fixed mucin in multi-well plates. PBS without bacteria was used as a control. Data represent means plus standard deviations of three replicates. * Statistically significant differences (*p* < 0.05) with respect to the control (PBS). The black dashed line represents the mean adhesion value of the negative control (PBS). Strain codes: IATA01, *Bifidobacterium animalis*; IATA35, *Bifidobacterium pseudocatenulatum*; IATA02, *Bifidobacterium longum*; IATA13, *Bifidobacterium bifidium*; IATA05, *Bifidobacterium longum*.

Another key factor in selecting the probiotic functionality of bacteria is their ability to adhere to mucin. The strains *B*. *bifidum* IATA13 (OD595 = 0.7) and IATA01 (OD595 = 1.63) were able to efficiently adhere to mucin compared to a PBS control (*p* < 0.05) (Figure 3). In fact, the strain *B*. *animalis* IATA01 showed a high capacity of adhesion. The rest of the strains remained within the response obtained by the PBS control.

### 3.3. In Vitro Colonic CF Microbiota Modulation

**Figure 4:**
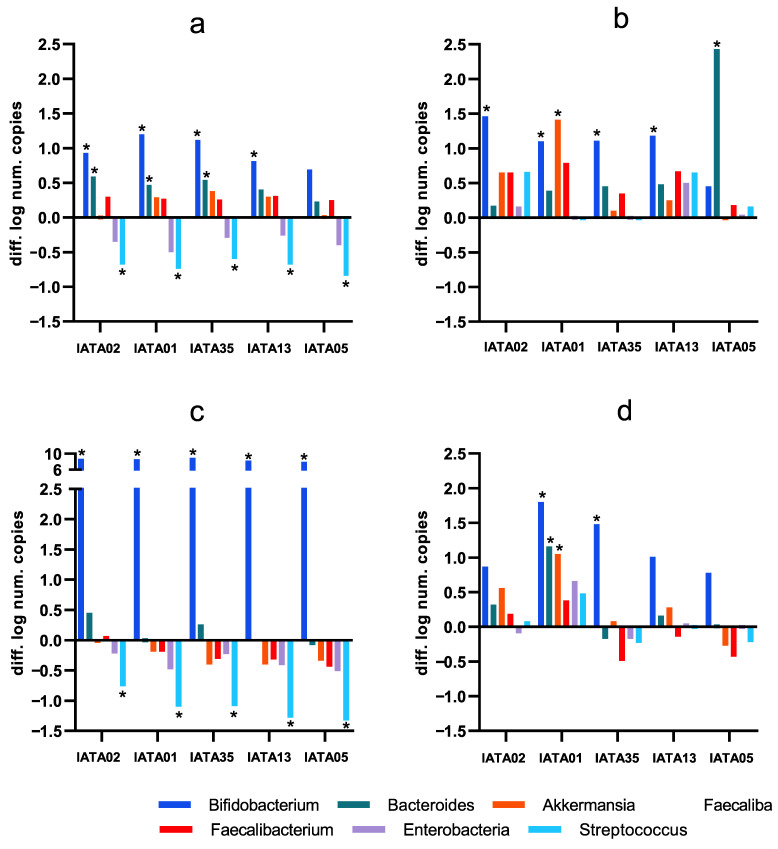
Changes in colonic microbiota as compared to the baseline fecal inocula composition induced by the simulation of colonic fermentation with the study strains (difference in the number of copies expressed as logarithm: after simulated colonic fermentation minus baseline faecal inoculum values, as determined by qPCR). (**a**) inoculum 1 (I1); (**b**) inoculum 2 (I2); (**c**) inoculum 3 (I3); (**d**) inoculum 4 (i4). I1–I4 denote the four individual patient inocula. * Statistically significant differences (*p* < 0.05) compared to baseline values. Strain codes: IATA01, *Bifidobacterium animalis*; IATA35, *Bifidobacterium pseudocatenulatum*; IATA02, *Bifidobacterium longum*; IATA13, *Bifidobacterium bifidium*; IATA05, *Bifidobacterium longum*.

Different effects of the *Bifidobacterium* strains were accounted depending on the type of fecal inoculum (Figure 4), as compared to the microbiota composition in the original inoculum from each subject (Supplementary Table S2). A common trend, though, was that most of the strains were able to induce a significant increase in the *Bifidobacterium* genus. This increase was especially relevant in inoculum 3 (I3), as the baseline values for this genus were relatively higher than in the other inocula. Another common observation was that all the strains were successful in reducing the populations of *Enterobacteriaceae* and *Streptococcus* in I1 and I3, while, in I2 and I4, the change in these groups was not relevant. Apart from *Bifidobacterium*, in I1, *Bacteroides* showed a significant increase in some of the cases. Focusing on I3, a decrease in beneficial groups, e.g., *Faecalibacterium prausnitzii* and *Akkermansia muciniphila*, was depicted for most of strains, although it was not detected as statistically significant. In contrast, these species, along with *Bacteroides*, were increased by most of the strains in I1 and I2, and only by *B*. *animalis* IATA01 in I4. Focusing on the exceptions, it is noticeable that *B*. *longum* IATA02 and *B*. *bifidum* IATA13 were the only strains that induced the growth of *Bacteroides* and *Streptococcus*, but this was only noted in I2. Another uncommon result was the growth of the *Bacteroides* group, which was relatively higher in I2 as induced by the strain *B*. *longum* IATA05, as it was the only bacterial group that showed a positive change in I3, apart from *Bifidobacterium* genus, which is related to *B*. *longum* IATA02 and *B*. *pseudocatenulatum* IATA35. Of note, *B*. *animalis* IATA 01 was the only strain that induced a significant increase in *Akkermansia muciniphila*, in the context of I2 and I4.

### 3.4. Colonic Metabolic Activity

**Figure 5:**
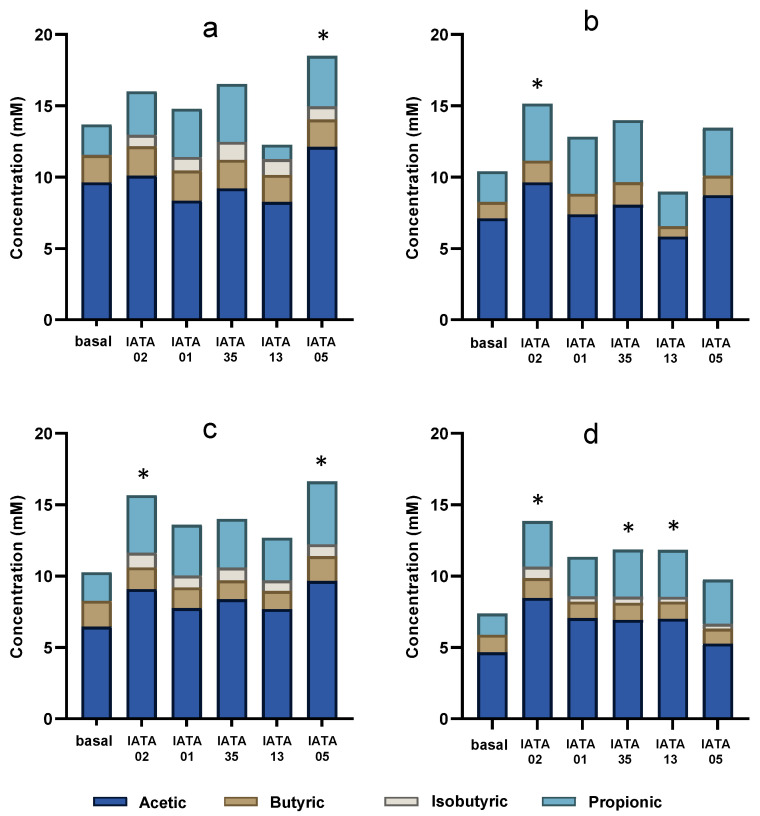
SCFAs produced after the administration of bifidobacterial strains during colonic fermentation in four faecal inocula. (**a**) inoculum 1 (I1); (**b**) inoculum 2 (I2); (**c**) inoculum 3 (I3); (**d**) inoculum 4 (i4). * Statistically significant differences (*p* < 0.05) compared to baseline values. Strain codes: IATA01, *Bifidobacterium animalis*; IATA35, *Bifidobacterium pseudocatenulatum*; IATA02, *Bifidobacterium longum*; IATA13, *Bifidobacterium bifidium*; IATA05, *Bifidobacterium longum*.

An analysis of SCFAs was performed as an indicator of modulation of metabolic activity of *Bifidobacterium* strains on gut microbiota of paediatric patients. An increase in the total SCFA produced by gut microbiota was observed in all inoculum environments with all the *Bifidobacterium* strains, as compared to baseline (gut microbiota samples without addition of *Bifidobacterium* strains) (Figure 5). In general, inocula I3 and I4 experienced the highest increase in SCFA production compared to their baseline levels. The most abundant SCFA was acetic acid, followed by propionic acid in all the inocula and for all the strains. However, the increase was only significant for the *B*. *longum* strain IATA05 in I1 (*p* = 0.0349) and I3 (*p* = 0.001); and in I2 (*p* = 0.022) and I3 (*p* = 0.004) for *B*. *longum* IATA02. In addition, focusing on I4, which had the overall lowest total SCFA among the four inocula, the most significant increase was achieved by *B*. *longum* IATA05 (*p* = 0.0012), *B*. *pseudocatenulatum* IATA35 (*p* = 0.0262) and *B*. *animalis* IATA01 (*p* = 0.0258).

When assessing the specific detected SCFAs (propionic, isobutyric, butanoic, and acetic acids), a common observation was the absence (or low concentration < 0.005 mM) of isobutyric acid in all the basal inocula, but its presence was detected in I1, I3, and I4, in variable proportions depending on the *Bifidobacterium* strain. Overall, the strains differentially modulated SCFA production, with the magnitude and profile of changes depending on the specific microbiota inoculum. Specifically, all the strains increased the level of isobutyric acid in all the inocula except I3 and *B*. *longum* IATA02 increased the acetic and propionic acids in I2 and the propionic, butyric, and isobutyric acids in I4.

## 4. Discussion

In the present study, different functional and probiotic capacities of novel isolated representative *Bifidobacterium* strains were assessed in the context of CF dysbiotic microbiota. Although several *Bifidobacterium* strains are used as probiotics [30], the five isolates from the mother–infant gut microbiota could offer an advantage in colonising the pediatric gut, as vertically transmitted bacteria are more stable colonisers and ecologically better adapted compared with non-maternal strains [31,32]. Some assessed parameters were related to strain survival in gastrointestinal tract conditions, capabilities of adhesion to the mucus layer (mucin adherence), and functional aspects such as diet carbohydrate utilisation, while others focused on the impact on colonic microbiota. Notably, this is the first study assessing the in vitro effect of *Bifidobacterium* strains on the colonic microbiota of children with CF. Furthermore, the characterization was designed to reflect key ecological and metabolic challenges regarding the distal colon in patients with CF, such as specific simulated gastrointestinal conditions and limited nutrient availability [13,14].

The results showed that the strains *B*. *pseudocatenulatum* IATA35, *B*. *animalis* IATA01, and *B*. *longum* IATA02 survived gastrointestinal digestion, but only *B*. *animalis* IATA01 adhered to mucin. Despite its lower carbohydrate fermentation capacity, *B*. *animalis* IATA01 induced the most beneficial changes in colonic microbiota. Of note, none of the tested strains utilised inulin or FOS in our assay, and we did not genotype β-fructofuranosidase/fructan loci. Inulin is a well-established prebiotic; thus, the absence of fructan utilisation is a limitation of the present strain set, especially considering that other isolates of *Bifidobacterium* species showed this capacity [33]. Nevertheless, probiotic effectiveness can arise via complementary mechanisms—mucin adhesion and niche occupation, antagonism of potential pathogens, and cross-feeding that enhances community-level SCFA production. Moreover, in pediatric CF, alternative glycans such as lactose derivatives, HMOs, and GOS may better match both the diet and the glycan capabilities of early-life bifidobacteria. Future work should include whole-genome sequencing to resolve CAZyme repertoires, targeted selection of fructan-utilising strains, and synbiotic designs pairing strains with matched prebiotics.

The study design follows some well-established methods for screening probiotic potential, as indicated by the FAO/WHO guidelines [34]. Evaluations such as survival in gastrointestinal conditions and mucin adherence are commonly conducted [27,35,36], while others assess antibiotic resistance [37] or pathogen inhibition capacity [38]. However, this study included some particularities for probiotic research. First, the use of a fluorescence staining technique (BacLight Live/Dead kit) to evaluate bacterial survival under simulated gastrointestinal conditions in the context of pediatric cystic fibrosis. This approach enabled the detection of both viable cells and viable but non-culturable (VBNC) cells. Some probiotic strains can exhibit a VBNC state under certain stress conditions; although they are unable to grow on standard culture media—a major limitation for assessing their proliferation—they can still retain key probiotic properties relevant to host interactions [39,40]. Then, the adhesion to mucin is a relevant test in the context of cystic fibrosis. Alterations in mucus composition and viscosity caused by defective bicarbonate transport in intestinal cells impact the intestinal environment [41]. Mucins, the structural glycoproteins of mucus, become abnormally concentrated and less hydrated, which leads to a thicker barrier that impairs clearance and facilitates pathogen colonization [41]. These changes not only contribute to gut dysbiosis and inflammation but also modify the interface where beneficial microbes interact with the host. Thus, a higher ability of adhesion to mucin would increase the colonization capacity of beneficial bacteria more effectively in this altered environment. Furthermore, few studies have examined a probiotic strain’s potential to modify colonic microbiota and metabolite production. Additionally, we assessed this effect in four different individuals within the framework of CF-induced dysbiosis. A comparable study analysed the impact of bacterial strains on colonic microbiota from feecal inocula of obese and normal-weight subjects, finding that probiotic addition did not induce major compositional changes apart from increases in the administered species [42]. In contrast, our study revealed significant reductions in potential pathogenic bacterial groups and increases in beneficial genera. Notably, the previous study did not assess genera beyond *Bifidobacterium* and *Lactobacillus* [42].

The specific impact on colonic microbiota varied by inoculum, highlighting individual-specific responses. In I1 and I3, all five *Bifidobacterium* strains reduced potentially pathogenic groups (enterobacteria and *Streptococcus*). However, no shared characteristics between I1 and I3 were identified apart from them being the oldest participants. Additionally, these two individuals exhibited the highest overall SCFA production. In I1, I2, and I4, at least one *Bifidobacterium* strain promoted the growth of bacterial groups beyond *Bifidobacterium*, whereas, in I3, only *Bifidobacterium* growth was observed, with the highest detected abundance among all cases. In I2, only positive or neutral growth effects were detected, and this inoculum exhibited the highest growth of *Bacteroides* and *Faecalibacterium*. Notably, I2 was the only individual without pancreatic insufficiency, a common CF condition that alters gastrointestinal parameters such as pH, nutrient absorption, and motility [13,14,15,16]. In I1, all strains enhanced beneficial bacterial groups while reducing potential pathogens. This participant was the only one undergoing CFTR modulator therapy, which can reverse the genetic defect underlying CF, improving gastrointestinal complications and potentially colonic microbiota dysbiosis [43]. These findings suggest that individual characteristics influence colonic microbiota modulation by probiotics, and that specific strains may be more effective depending on the basal microbiota profile.

Among the study strains, *B*. *animalis* IATA01 emerged as the most promising candidate for microbiota improvement and future probiotic applications. In I1, its effects were comparable to those of other strains; in I2, it induced the highest *Akkermansia* and *Faecalibacterium* growth; in I3, its impact was similar to that of the other strains; and in I4, it promoted the highest growth of *Bacteroides*, *Bifidobacterium*, *Akkermansia*, and *Faecalibacterium*.

*B*. *animalis* IATA01 exhibited high mucin adhesion, a key characteristic for probiotic bacteria that enhances persistence in the gastrointestinal tract, triggers host responses, and prevents pathogen colonisation by competing for binding sites [44,45,46]. A previous study identified a *B*. *breve* strain with the highest digestion survival and mucin adherence, but these properties were not assessed in *B*. *animalis* [35]. Harata et al. (2021) similarly found that *B*. *animalis* strains exhibited the highest mucin adherence [47]. Most probiotic screening studies focus on *B*. *infantis*, *B*. *breve*, *B*. *adolescentis*, or *B*. *longum*, which limits direct comparisons with *B*. *animalis* [48]. Likewise, *B*. *pseudocatenulatum*, which also demonstrated promising results, has been scarcely studied in this context. Mucin adhesion in *Bifidobacterium* is strain-specific, with higher adherence observed in *B*. *longum* [44,49] and *B*. *pseudolongum* subsp. *pseudolongum* [45]. Various adhesins contribute to this trait, including DnaK in *B*. *animalis* subsp. *lactis* Bi-0744 and exopolysaccharides in *B*. *animalis* IPLA-R145. *B*. *animalis* IATA01′s strong mucin-binding ability may be particularly relevant in CF, where antibiotic use increases the risk of pathogenic colonisation [10,50]. Additionally, probiotic adhesion facilitates host interactions. *B*. *animalis* IATA01 metabolised glucose, lactose, and ribose, which suggests potential for cross-feeding with other colonic bacteria, enhancing SCFA production [50,51].

Regarding SCFAs, this study demonstrated that the selected *Bifidobacterium* strains modulated the production of SCFAs, particularly acetate, propionate, and isobutyric acids, while changes in butyric acid were minor during colonic fermentation. Although acetic acid is the main SCFA produced by *Bifidobacterium* strains, these results highlight the modulatory effect of these strains on composition and functionality, suggesting cross-feeding interactions that enhance SCFA output. These metabolites are central in host interactions since they have been associated with improved mucosal integrity, reduced inflammation, and immune and metabolic modulations [52]. These findings are relevant in the CF context, as it has been described that children without CF had higher levels of acetate, butyrate, and propionate compared to a similar cohort of children with CF [2,53].

This exploratory framework (strain functional characterization including in vitro modulation of gut microbiota), applied to a larger cohort, could serve as a clinical tool for identifying suitable probiotics based on individual characteristics. Despite its relevance, this study has several limitations. The colonic fermentation medium contained only basal nutrients, which may have constrained bacterial growth compared to a medium supplemented with specific substrates. Other limitations include the small number and heterogenicity of faecal inocula, the static in vitro fermentation model, and the use of qPCR rather than 16S sequencing for microbiota analysis. Nonetheless, qPCR was deemed appropriate given this study’s screening purpose, allowing reliable and fast quantification of relevant bacterial groups. Another limitation is that qPCR does not distinguish between culturable, viable but nonculturable (VBNC), or dead cells. Of note, the VBCN state is characterized by the loss of culturability of microbial cells on/in nutrient media that normally support their growth, while maintaining metabolic activity [54]. Moreover, the ability of probiotics to impact VBNC pathogens may still be ecologically relevant, since VBNC cells can resuscitate under favourable conditions and contribute to infection, and future studies using complementary approaches such as viability-qPCR or biocide efficiency that apply culture-based methods could further resolve this issue [54]. Whole-genome sequencing and further analyses are necessary for deeper strain identification, safety evaluation, and elucidation of probiotic–host interaction mechanisms.

Conversely, this study presents notable strengths. Bacterial viability and gastrointestinal survival assessments were conducted stepwise using an appropriate in vitro digestion model (enzyme combination, duration, temperature, agitation, pH values), in contrast to previous studies that evaluated only gastric pH and bile salt concentrations [49]. Additionally, this screening specifically examined microbial modulation in children with CF, supporting probiotic-based treatments tailored to paediatric patients with CF. Another key contribution is the assessment of *Bifidobacterium* strains derived from the maternal–infant environment, a crucial source for this genus, with potential applications in probiotic development. Finally, by focusing on CF, a condition characterised by gastrointestinal dysfunction and altered microbiota with significant reduction in the *Bifidobacterium* genus, this study highlights opportunities for probiotic interventions to support gastrointestinal health. Thus, this study analysed the carbohydrate capability of *Bifidobacterium* strains to assess their bifidogenic properties and to allow their establishment and functionality in the cystic fibrosis environment, supporting their use in targeted synbiotic interventions.

In conclusion, this study suggests the strain-dependent activity of *Bifidobacterium* and evidence that the potential probiotic effects vary according to the initial microbial community, emphasizing the importance of individual-specific responses.

## Figures and Tables

**Table 1:** Characteristics of the paediatric patients with cystic fibrosis who donated fecal samples to obtain the inoculum (I) to simulate colonic fermentation, at the moment of sample collection.

	I1	I2	I3	I4
Age (years)	17	8	10	6
Gender	Female	Female	Male	Female
CFTR Mutation	F508del/F508del	F508de/D110H	F508de/R347P	F508del/c.2908 + 1G > A
Pancreatic insufficiency and PERT *	Yes	No	Yes	Yes
Antibiotics	No	Yes	Yes	Yes
CFTR ** modulator therapy	Yes	No	No	No
Nasopharyngeal exudate	Normal microbiota	*S*. *aureus*	*S*. *aureus*	*S*. *aureus*

* PERT, pancreatic enzyme replacement therapy. ** Cystic fibrosis transmembrane conductance regulator protein.

**Table 2:** Taxonomic identification of novel *Bifidobacterium* strains obtained from the maternal–infant environment.

Isolate Code	Taxonomic Identification (rRNA 16S) *	Source
IATA01	*Bifidobacterium animalis*	Adult feces (mother)
IATA35	*Bifidobacterium pseudocatenulatum*	Adult feces (mother)
IATA02	*Bifidobacterium longum*	Adult feces (mother)
IATA13	*Bifidobacterium bifidum*	Adult feces (mother)
IATA05	*Bifidobacterium longum*	Infant feces (baby)

* Taxonomic assignment is limited to the species level based on partial 16S rRNA gene sequencing.

## Data Availability

The datasets generated during and/or analysed during the current study are available from the corresponding author on reasonable request.

## Supplementary Materials

The following supporting information can be downloaded at: https://www.mdpi.com/article/10.3390/microorganisms13112523/s1, Table S1. Primers used in this study; Table S2. Bacterial groups in the original inoculum samples of each patient (I1–I4) expressed as number of copies/mL.
