# Supplementary material for: Exploratory In Vitro Evaluation of Maternal–Infant Bifidobacterium Strains for Microbiota Modulation in a Pediatric Cystic Fibrosis Context
Source: Microorganisms. 2025 Nov 2;13(11):2523. doi: 10.3390/microorganisms13112523 (PMC12654849; doi:10.3390/microorganisms13112523)
Supplement: Supplementary file 1 [file microorganisms-13-02523-s001.zip › microorganisms-3809936-supplementary.pdf]

Table S1. Primers used in this study

Target	Primers	Sequence from 5′ to 3′
Total bacteria	16S_U515F	GTGCCAGCMGCCGCGGTAA
	16S_U789R	GCGTGGACTACCAGGGTATCT
Bifidobacterium genus	Bifido5’	GATTCTGGCTCAGGATGAACGC
	Bifido3’	CTGATAGGACGCGACCCCAT
Bacteroides genus	Bact5'	GGTGTCGGCTTAAGTGCCAT
	Bact3'	CGGA(C/T)GTAAGGGCCGTGC
F. prausnitzii	Fprau 07	CCATGAATTGCCTTCAAAACTGTT'
	Fprau 02	GAGCCTCAGCGTCAGTTGGT'
A. muciniphila	akkerF	CAGCACGTGAAGGTGGGGAC
	akkerR	CCTTGCGGTTGGCTTCAGAT'
Enterobacteria group	EnteF	CATTGACGTTACCCGCAGAAGAAGC
	EnteR	CTCTACGAGACTCAAGCTTGC
Streptococcus genus	Strep-1	GTACAGTTGCTTCAGGACGTATC
	Strep-2	ACGTTCGATTTCATCACGGTT

Table S2. Bacterial groups in the original inoculum samples of each patient (I1-I4) expressed as number of copies/mL

Bacterial groups	I1	I2	I3	I4
Total bacteria	3,83E+09	4,81E+09	6,80E+09	1,14E+10
Bifidobacterium genus	2,35E+07	3,27E+07	0,00E+00	2,81E+07
Bacteroides genus	6,31E+08	1,55E+09	1,86E+09	1,71E+09
Akkermansia muciniphila	1,80E+07	1,45E+06	1,23E+04	6,31E+03
Faecalibacterium prausnitzii	6,80E+02	2,01E+05	7,80E+02	2,55E+04
Enterobacteria group	1,57E+09	6,99E+08	1,14E+09	1,29E+09
Streptococcus genus	1,63E+03	1,69E+02	5,65E+03	8,65E+02
